# Observation on the application effect of chest physical therapy based on lung ultrasound signs in the respiratory management of mechanically ventilated patients

**DOI:** 10.3389/fmed.2025.1708677

**Published:** 2025-12-03

**Authors:** Xiuqin Chen, Yongrui Wu, Li Zhang, Mengqi Sun, Zhengwei Li, Hui Fang

**Affiliations:** 1 Intensive Care Unit, Taihe County People’s Hospital, Fuyang, Anhui, China; 2 Medical School, Fuyang Normal University, Fuyang, Anhui, China

**Keywords:** mechanical ventilation, chest physical therapy, lung ultrasound, diaphragmatic function, oxygenation index

## Abstract

**Objective:**

This study aimed to explore the efficacy of chest physical therapy guided by lung ultrasound in the respiratory management of patients with invasive mechanical ventilation.

**Methods:**

A prospective study design was adopted. A total of 100 patients with invasive mechanical ventilation admitted to the intensive care unit (ICU) of the hospital from January 2024 to March 2025 were selected and divided into an observation group and a control group, with 50 cases in each group, by the random number table method. The control group received chest physical therapy guided by conventional auscultation combined with chest X-ray, while the observation group received chest physical therapy guided by lung ultrasound. The lung ultrasound scores (LUS), diaphragmatic function parameters (displacement and thickening rate), and blood gas indicators of the two groups were compared after 3 days and 7 days of treatment. The duration of mechanical ventilation, ICU stay time, and complication incidence were also recorded.

**Results:**

After 3 days and 7 days of treatment, the LUS of the observation group were significantly lower than that of the control group (*p* < 0.05). In terms of diaphragmatic function, there was no significant difference between the two groups after 3 days of treatment (*p* > 0.05); the observation group demonstrated significantly greater diaphragmatic displacement and thickening rate than the control group after 7 days (*p* < 0.05). Blood gas analysis showed that the partial pressure of oxygen and oxygenation index of the observation group were increased compared with the control group, and the partial pressure of carbon dioxide was decreased (all *p* < 0.05). The duration of mechanical ventilation and intensive care unit (ICU) stay in the observation group was shorter, and the total incidence of complications (e.g., ventilator-associated pneumonia) was 14% lower (all *p* < 0.05).

**Conclusion:**

Chest physical therapy guided by lung ultrasound can effectively improve pulmonary ventilation and diaphragmatic function in patients on mechanical ventilation, enhance oxygenation efficiency, shorten the treatment cycle, and reduce the risk of complications. These findings demonstrate its significant clinical value and promote its wider application.

## 1 Introduction

Mechanical ventilation is the primary therapeutic approach employed in the clinical management of patients experiencing dyspnea. Its principal functions include maintaining airway patency, enhancing ventilation and oxygenation, and preventing systemic hypoxia. While mechanical ventilation facilitates the clinical treatment of underlying pathologies, prolonged use is associated with complications such as atelectasis, pulmonary infections, and diaphragmatic dysfunction (1, 2), which can result in extended hospital stays and increased mortality rates. Chest physiotherapy (CPT) is a widely utilized intervention aimed at mitigating complications in patients undergoing mechanical ventilation. CPT encompasses techniques such as chest percussion, active cycle breathing techniques, inspiratory muscle training, manual lung inflation, vibration for sputum clearance, and postural drainage. These methods collectively contribute to improved pulmonary function, thereby potentially reducing the duration of mechanical ventilation and the risk of associated complications (3, 4). However, the effectiveness of auscultation and chest X-rays in guiding chest physiotherapy is constrained by body position, which impedes accurate and timely lung condition assessment and impacts treatment outcomes. Functioning as a “visual stethoscope,” lung ultrasound enables real-time imaging by identifying characteristic signs associated with changes in the lung air-to-fluid ratio. LUS offers advantages such as the absence of radiation, bedside applicability, and dynamic monitoring capability (5). This study included 100 ICU patients receiving invasive mechanical ventilation between January 2024 and March 2025 to evaluate the value of chest physiotherapy guided by lung ultrasound signs. A prospective study was conducted to compare this method with conventional chest physiotherapy, and the findings are presented below.

## 2 Subjects and methods

### 2.1 Research subjects

A total of 100 patients who were admitted to the ICU and received invasive mechanical ventilation treatment from January 2024 to March 2025 were included in this study. Using the random number table method, the subjects were randomly assigned to a control group or an observation group, with 50 cases in each.

The inclusion criteria were as follows: (1) adult patients (aged ≥18 years); (2) undergoing invasive mechanical ventilation treatment in the ICU, with the expected duration of mechanical ventilation and ICU stay ≥72 h; (3) hemodynamic stability; (4) no contraindications for physical therapy (such as coagulation disorders, rib injuries, *etc*.).

The exclusion criteria were as follows: (1) limited lung ultrasound examination (complicated with severe complications such as tension pneumothorax or mediastinal emphysema); (2) intracranial pressure ≥20 cm H_2_O; (3) pulmonary embolism; (4) voluntarily giving up treatment, being discharged from the hospital, or dying. With the significance level set at *α* = 0.05, statistical power at 0.8, and an effect size of 0.5, the sample size was estimated using GPower 3.1 software. The results indicated that 45 subjects were required for each of the observation and control groups. To account for potential dropouts, loss to follow-up, and mortality, a total of 100 patients were enrolled. Allocation concealment was implemented for the patients, ultrasonographers, and statisticians involved in the study. Using a random number table, subjects were assigned to one of two groups based on their ICU admission order (odd or even numbers). Those with odd numbers were allocated to the control group, while those with even numbers were assigned to the observation group, with 50 patients in each group, respectively.

The research was approved by the hospital’s ethics committee (no. 2023-19), and the informed consent forms were signed by the patients’ family members. All the research subjects underwent comprehensive lung examinations, such as auscultation, X-ray, and CT, upon admission to the department.

### 2.2 Research methods

#### 2.2.1 Establishment of the research group

The research team was composed of six members: two nurses specializing in intensive care, one intensive-care physician, one ultrasound department professional, one respiratory therapist, and one rehabilitation therapist. Each member possessed the requisite qualification certificates. Notably, one of the intensive-care specialty nurses had completed an advanced training course in intensive-care ultrasound nursing. The responsibility for lung ultrasound scans fell to a designated nurse, who collaborated with the intensive care physician. CPT was implemented by the respiratory and rehabilitation therapists with nursing support. A dedicated intensive care nurse collected the baseline data and evaluation metrics.

#### 2.2.2 Control group

Upon ICU admission and within 24 h of initiating invasive mechanical ventilation, patients’ pulmonary conditions were assessed by chest X-ray, CT, or auscultation. Chest physiotherapy comprises assessment, aerosol inhalation, postural drainage, sputum expulsion using a vibratory sputum excretion device, and sputum suction. Based on this assessment, routine chest physiotherapy was then implemented. Specific interventions included: (1) maintaining a 30° semi-recumbent position with repositioning every 2 h; (2) performing manual percussion and vibration to facilitate sputum clearance; and (3) administering instrument-assisted sputum excretion three times daily for 15 min each session. The intervention was immediately terminated if vital signs deviated by more than 20% from baseline.

#### 2.2.3 Observation group

The observation group received LUS-guided care from specially trained intensive care nurses in addition to the standard treatment given to the control group. These nurses regularly monitored the lungs and diaphragm using ultrasound technology, communicated the findings to physicians and rehabilitation therapists, and tailored chest physiotherapy strategies based on ultrasound results.

##### 2.2.3.1 Chest physiotherapy

(1) Pre-procedure: Review the patient’s records and assess their condition. Provide explanations to conscious patients. (2) Aerosol inhalation: Dilute the medication with 0.9% sodium chloride solution or sterile water for injection to a volume of 4–5 mL. Adjust the oxygen flow rate to 6–8 L/min and connect the circuit to the Y-piece inlet of the ventilator. (3) Postural drainage: Perform postural drainage only when the patient’s vital signs are stable. Each session should not exceed 15 min. (4) Sputum expulsion using a vibratory sputum excretion device: Use the device at a frequency of 20–30 CPS, once every 6 h, for 15–20 min per session. With the patient in a semi-Fowler’s or lateral sitting position, the assigned nurse holds the device handle and performs percussion from the bottom to the top and from the outside to the inside on the patient’s back and chest. (5) Sputum suction: Perform suction as needed based on lung auscultation findings. Each suction session should not exceed 15 s.

##### 2.2.3.2 Lung ultrasound assessment

Each day before treatment, two critical care ultrasound nurses used bedside ultrasound technology to systematically evaluate both lung fields (divided into 12 examination areas) and diaphragm function, with a focus on identifying characteristic imaging changes, such as B-lines, lung consolidation or atelectasis, and pleural line abnormalities. Based on the distinct manifestations observed in the lung ultrasound, appropriate nursing interventions were implemented: 1. In cases where the lung ultrasound revealed a pleural sliding sign and A-lines, confirming adequate ventilation, optimal position management was ensured by maintaining the head of the bed elevated at 30°, and patients were assisted in repositioning every 2 h. 2. The detection of B3-lines and/or B7-lines suggested an increase in pulmonary interstitial water content. This could result from factors such as sputum retention, elevated airway resistance, and thickening of the respiratory membrane, potentially leading to ventilation or gas-exchange dysfunction. In such cases, airway management was intensified through high-frequency vibration to facilitate sputum clearance, supplemented by active or passive breathing exercises to enhance respiratory function. 3. Upon detection of a shred sign or tissue-like sign, indicating consolidation/atelectasis and impaired ventilation, a standardized protocol was initiated. This protocol included airway suction, postural management, vibration sputum excretion, and breathing exercises to loosen secretions and recruit alveoli, complemented by early mobilization to mitigate weakness and improve pulmonary function. Nurses dynamically adapted these strategies based on ongoing ultrasound monitoring.

### 2.3 Observation indicators and evaluation criteria

#### 2.3.1 Observation indicators

1. Changes in lung ultrasound (LUS) scores, diaphragmatic parameters (displacement and thickening rate), and arterial blood gas analysis, measured at baseline (pre-intervention) and on days 3 and 7 post-intervention.
2. Secondary indicators: We assessed the duration of mechanical ventilation, length of stay in the ICU, and the incidence of associated complications, such as atelectasis and ventilator-associated pneumonia, in patients from both groups.

#### 2.3.2 Evaluation criteria for lung ultrasound

##### 2.3.2.1 Quantitative evaluation of LUS

**Figure 1:**
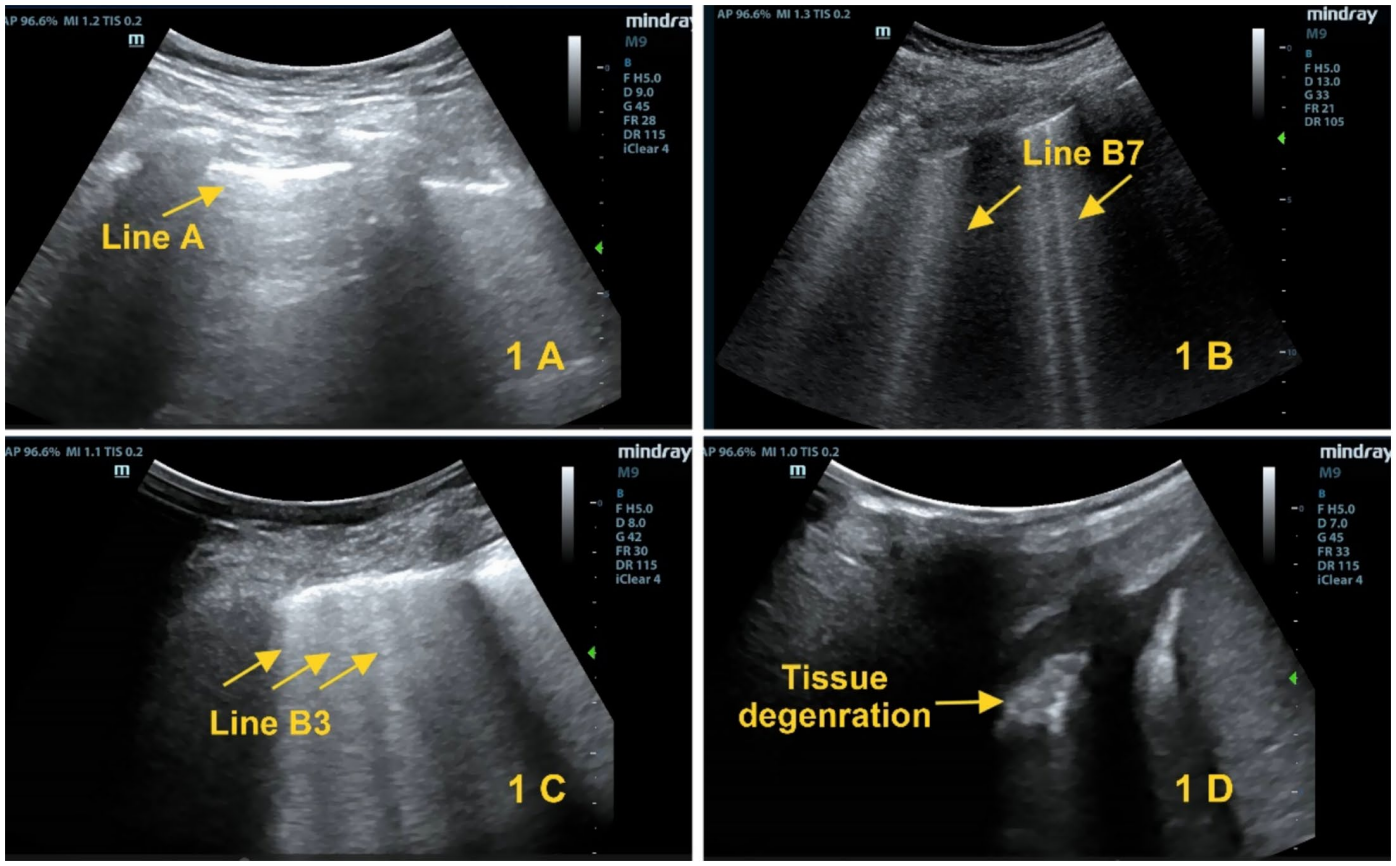
Lung ultrasound scoring criteria. **(A)** Score 0: A-lines (yellow arrows). **(B)** Score 1: B1 lines (yellow arrows). **(C)** Score 2: B2 lines (yellow arrows). **(D)** Score 3: Tissue-like sign (yellow arrows).

(1) Score 0: Pleural sliding sign with A-line visualization (Figure 1A); (2) Score 1: B7-line sign (B-line spacing of 7 mm, Figure 1B); (3) Score 2: B3-line sign (B-line spacing of 3 mm, Figure 1C); (4) Score 3: Shred sign/tissue-like change (Figure 1D). A 12-lung-zone (6 zones on each side) scanning method is used, and the total score ranges from 0 to 36 points (6).

##### 2.3.2.2 Detection of diaphragmatic function

(1) Displacement was measured at the standardized costal arch position on the anterior axillary line. It was defined as the difference in diaphragmatic position between the end of expiration and the end of inspiration during the respiratory cycle; (2) Thickening rate calculation: With the patient positioned supine, a cross-sectional view is obtained at the 8th–10th intercostal spaces along the mid-axillary line. The thickening rate is then calculated using the formula: [(end-inspiratory thickness − end-expiratory thickness)/end-expiratory thickness] × 100%.

##### 2.3.2.3 Blood gas indicators

Arterial blood samples are collected from fasting patients in the early morning prior to intervention, on day 3, and on day 7 after intervention for blood gas analysis. The parameters measured include the inspired oxygen fraction (FiO_2_), partial pressure of arterial oxygen (PaO_2_), and partial pressure of arterial carbon dioxide (PaCO_2_). The PaO_2_/FiO_2_ value serves as the oxygenation index.

### 2.4 Data collection and quality control methods

A data collection form developed for ICU patients was used to record the baseline data, such as age, gender, acute physiology and chronic health evaluation II (APACHE II) score, and primary diagnosis. The collection was completed by intensive care specialty nurses within 24 h after the enrollment of the patient. Before the intervention, trained intensive care ultrasound nurses assessed and recorded the patients’ LUS, diaphragmatic displacement, and thickening rate. After 2 h of the intervention, lung ultrasound examinations were repeated for patients in both groups, and the corresponding parameters were documented. The oxygenation index is derived from arterial blood gas analysis conducted daily at 07:00. Upon the patient’s transfer out of the ICU, data on mechanical ventilation duration, ICU length of stay, and the incidence of related complications, such as atelectasis and ventilator-associated pneumonia, are recorded.

To ensure consistency in the evaluation process, each lung ultrasound assessment, along with the measurement of diaphragmatic movement amplitude and the calculation of thickness variation rate, is conducted collaboratively by two nurses. If there are differences in image interpretation, a doctor is invited to assist in the determination to reduce operator bias. All data are subjected to a double-check by team members before being entered into an Excel spreadsheet.

### 2.5 Statistical methods

Data analysis is performed using the SPSS 26.0 statistical software. Following a normality test, measurement data conforming to a normal distribution are presented as mean ± standard deviation ( $\bar{x}$ *±* s). The independent-samples t-test is used for comparison between groups. Repeated measures data were analyzed using repeated measures analysis of variance. Counting data are described by frequency (percentage), and the *χ*^2^ test is used for analyzing the differences between groups. A *p* < 0.05 is considered indicative of statistical significance.

## 3 Results

### 3.1 Comparison of general data between the two groups

There was no statistically significant difference in the general data between the two groups (*p* > 0.05), indicating that the two groups were comparable (Table 1).

**Table 1:** Comparison of general data between the two groups.

Groups	Observation group (*n* = 50)	Control group (*n* = 50)	$\chi^{2}/t$	*p*
Gender [*n* (%)]			0.750	0.387
Male	29 (58.00)	27 (54.00)		
Female	21 (42.00)	23 (46.00)		
Age (Years, $\bar{x}$ *±* s)	63.06 ± 5.91	62.74 ± 5.86	0.272	0.786
APACHEII score (points, $\bar{x}$ *±* s)	17.73 ± 2.26	17.69 ± 2.28	0.088	0.930
Type of Shock [*n* (%)]			1.521	0.957
Severe pneumonia	17 (34.00)	19 (38.00)		
COPD	12 (24.00)	9 (18.00)		
Cerebrovascular disease	10 (20.00)	11 (22.00)		
After abdominal surgery	6 (12.00)	4 (8.00)		
Sepsis	2 (4.00)	2 (4.00)		
Multiple injuries	2 (4.00)	3 (6.00)		
Others	1 (2.00)	2 (4.00)		

### 3.2 Comparison of LUS between the two groups

No significant difference in LUS was found between the two groups before treatment (*p* > 0.05). In the observation group, however, LUS decreased significantly on days 3 and 7 compared to both baseline (within-group, *p* < 0.05) and the control group (between-group, *p* < 0.05), as detailed in Table 2.

**Table 2:** Comparison of LUS between two groups ( $\bar{x}$ *±* s).

Groups	Before treatment	Treatment for 3 days	Treatment for 7 days
Control group (*n* = 50)	20.89 ± 5.13	17.32 ± 3.42	9.78 ± 2.21
Observation group (*n* = 50)	21.23 ± 5.16	15.74 ± 3.38	8.86 ± 2.17
*t*	0.330	2.324	2.100
*p*	0.742	0.022	0.038

*F*(between-group) = 17.149, *p* < 0.001; *F*(time) = 166.743, *p* < 0.001; *F*(interaction) = 2.437, *p* = 0.049.

### 3.3 Comparison of diaphragmatic displacement and diaphragmatic thickening rate between the two groups

There was no statistical difference in diaphragmatic displacement and thickening rate between the two groups at the initial stage of treatment (on day 3). During the treatment on the 7th day, the diaphragmatic displacement distance in the observation group increased compared with the baseline value, and the thickening rate was significantly improved. The improvement amplitude in the observation group was significantly higher than that in the control group (*p* < 0.05). The specific data are shown in Table 3.

**Table 3:** Comparison of diaphragm displacement and diaphragm thickening rate between the two groups ( $\bar{x}$ *±* s).

Groups	Diaphragmatic displacement (mm)			Diaphragmatic thickening rate (%)		
	Before treatment	Treatment for 3 days	Treatment for 7 days	Before treatment	Treatment for 3 days	Treatment for 7 days
Control group (*n* = 50)	11.63 ± 2.08	11.56 ± 2.4	12.58 ± 1.9	29.20 ± 4.61	30.39 ± 5.18	35.62 ± 4.93
Observation group (*n* = 50)	11.67 ± 2.07	11.59 ± 2.5	13.41 ± 2.1	29.12 ± 4.57	30.64 ± 5.13	38.91 ± 5.26
*t*	0.096	0.060	2.038	0.087	0.243	3.227
*p*	0.923	0.952	0.044	0.931	0.809	0.002

For diaphragmatic displacement: *F*(between-group) = 7.229, *p* = 0.009; *F*(time) = 43.499, *p* < 0.001; *F*(interaction) = 3.899, *p* = 0.005. For diaphragmatic thickening fraction: *F*(between-group) = 7.598, *p* = 0.007; *F*(time) = 19.208, *p* < 0.001; *F*(interaction) = 2.606, *p* = 0.039.

### 3.4 Comparison of blood gas index levels between the two groups

Blood gas analysis showed that the oxygenation indexes in the observation group had a better improvement trend. On the 7th day, the PaO_2_ and OI index were significantly increased, and at the same time, the PCO_2_ was maintained at a normal or lower level. The differences between the two groups were all statistically significant (*p* < 0.05) (see Table 4).

**Table 4:** Comparison of blood gas index levels between two groups ( $\bar{x}$ *±* s).

Groups	PaO_2_			PCO_2_			OI		
	Before treatment	Treatment for 3 days	Treatment for 7 days	Before treatment	Treatment for 3 days	Treatment for 7 days	Before treatment	Treatment for 3 days	Treatment for 7 days
Control group (*n* = 50)	56.34 ± 6.71	65.91 ± 6.70^a^	88.68 ± 6.51^b^	63.91 ± 5.64	52.61 ± 4.79^a^	44.57 ± 4.62^b^	183.84 ± 37.90	277.16 ± 39.71^a^	312.49 ± 41.77^b^
Observation group (*n* = 50)	56.62 ± 6.58	65.59 ± 6.65^a^	92.36 ± 6.44^b^	62.38 ± 5.69	49.56 ± 4.83^a^	41.94 ± 4.70^b^	195.78 ± 38.62	294.55 ± 40.63^a^	331.84 ± 43.25^b^
*t*	0.211	0.240	2.842	0.468	3.171	2.822	0.128	2.159	2.276
*p*	0.834	0.811	0.006	0.641	0.002	0.006	0.898	0.033	0.025

Comparison within the group before treatment, ^a^*p* > 0.05, ^b^*p* < 0.05.

### 3.5 Comparison of mechanical ventilation time, ICU stay time, and complications between the two groups

In the observation group, both the duration of mechanical ventilation and the ICU stay time were significantly shortened, and the incidence of complications was reduced. All the differences reached a statistically significant level (*p* < 0.05) (see Table 5).

**Table 5:** Comparison of mechanical ventilation time, ICU stay time, and complications between the two groups.

Groups	Mechanical ventilation time (d, $\bar{x}$ *±* s)	ICU stay time (d, $\bar{x}$ *±* s)	Complications			
			Atelectasis	VAP	DVT	Occur frequently
Control group (*n* = 50)	8.25 ± 1.97	13.67 ± 3.08	3 (6.00)	4 (8.00)	3 (6.00)	10 (20.00)
Observation group (*n* = 50)	7.03 ± 2.06	11.62 ± 3.15	1 (2.00)	1 (2.00)	1 (2.00)	3 (6.00)
$\chi^{2}/t$	3.027	4.189				4.332
*p*	0.003	0.000				0.037

## 4 Discussion

### 4.1 Implementing CPT under the guidance of lung ultrasound can improve the lung conditions of patients with invasive mechanical ventilation

Mechanical ventilation is a critical life-support intervention used in over 70% of patients in the ICU (7). However, prolonged mechanical ventilation carries the risk of complications such as pulmonary consolidation, atelectasis, and ventilator-associated lung injury (1), which represent major challenges in clinical practice. CPT has been demonstrated as an effective method to facilitate airway secretion clearance, enhance lung compliance, improve oxygenation, and decrease the incidence and mortality rates of pulmonary infections and ventilator-associated pneumonia (1, 8). In recent years, lung ultrasound has gained widespread application across various disciplines, including ICU pulmonary rehabilitation, due to its non-invasive, dynamic, and repeatable nature, allowing for bedside visual assessment of lung lesions (5). The World Interactive Network Focused on Critical Ultrasound (WINFOCUS) identified lung ultrasound as a crucial tool in respiratory management, with its imaging signs reflecting the pathophysiological changes occurring within the lungs (9). The twelve-zone ultrasound scanning method, based on the anatomical segmentation of the chest, enables objective assessment of pulmonary function through serial score changes. This approach facilitates personalized respiratory management and evaluates the efficacy of interventions. Lung ultrasound-guided respiratory management employs ultrasound scores as a quantitative metric, enabling the clear identification of conditions such as pleural effusion, pulmonary consolidation, and atelectasis. A higher score correlates with reduced gas content in the lungs and more severe pulmonary lesions (10). With real-time ultrasound monitoring, nurses can precisely ascertain the location and extent of lesions. Compared to standard nursing practices, this method is more conducive to developing scientifically grounded, precise, and individualized respiratory management strategies, as well as enabling dynamic evaluation of the treatment process (11, 12). By systematically implementing interventions such as postural drainage, respiratory muscle training, and lung recruitment, the functionality of respiratory muscles can be significantly enhanced, oxygenation improved, and hypoxic damage to lung tissue mitigated, thereby maintaining respiratory function stability. The findings of this study demonstrated a reduction in lung ultrasound scores in both groups following intervention; however, the scores in the observation group on the third and seventh days were notably lower than those in the control group (*p* < 0.05). This suggests that quantitatively guiding the implementation of CPT based on lung ultrasound scores facilitates timely adjustments in treatment and care focus, as well as the evaluation of therapeutic efficacy, thereby more effectively improving the pulmonary conditions of patients on mechanical ventilation. These results are consistent with the findings of Li et al. (13) regarding pulmonary rehabilitation training for patients undergoing open abdominal surgery under general anesthesia, as well as the results of Liu et al. (14) concerning ultrasound-guided lung recruitment.

### 4.2 Implementing CPT under the guidance of lung ultrasound can improve the diaphragmatic function of patients with invasive mechanical ventilation

Lung ultrasound-guided CPT has been shown to enhance diaphragmatic function in ICU patients undergoing mechanical ventilation. The diaphragm, as the primary respiratory muscle, is integral to the regulation of ventilation. Prolonged mechanical ventilation may result in diaphragmatic disuse, leading to fiber atrophy and diminished remodeling capacity (15). In addition, ultrasound is frequently employed to assess diaphragmatic function, with key indicators including diaphragmatic mobility, thickening fraction, and displacement degree (16, 17). Research indicates that the prevalence of diaphragmatic dysfunction is notably high among critically ill patients, affecting up to 64% upon ICU admission, and it is a significant factor influencing successful weaning from mechanical ventilation (18). The extent of diaphragmatic displacement is indicative of positional changes during respiration. CPT has demonstrated efficacy in enhancing both diaphragmatic and respiratory functions in patients receiving mechanical ventilation. In this study, CPT intervention, guided by lung ultrasound, was implemented. The findings indicated that on the seventh day of intervention, the diaphragmatic displacement and thickening rate in the observation group were significantly greater than those in the control group (*p* < 0.05), suggesting that this intervention effectively enhances diaphragmatic function. The potential mechanism underlying this effect may involve personalized CPT under real-time ultrasound guidance, which can augment diaphragmatic blood perfusion, improve oxygenation, and mitigate oxidative stress injury through targeted respiratory muscle training (19). Additionally, it may prevent diaphragmatic disuse and its associated functional decline (20). These results are consistent with the findings of Chen et al. (21) and Liu X et al. (22), further corroborating the beneficial impact of chest physical therapy on the diaphragmatic function of patients undergoing mechanical ventilation.

### 4.3 Implementing CPT under the guidance of lung ultrasound can improve the oxygenation and ventilation status of patients with invasive mechanical ventilation

The oxygenation index serves as a critical parameter for assessing pulmonary gas exchange function and the efficiency of oxygen delivery, thereby reflecting the lungs’ capacity for oxygen exchange. A reduction in this index suggests pulmonary respiratory dysfunction and may elevate the risk of hypoxemia and respiratory failure. Lung ultrasound-guided respiratory management allows for the dynamic, real-time monitoring of pulmonary lesions. Based on ultrasound scoring, appropriate CPT strategies can be selected to progressively enhance lung function. Concurrently, targeted nursing interventions can be implemented by identifying severely affected areas, facilitating timely and precise interventions, minimizing iatrogenic injuries, and improving pulmonary ventilation efficiency (23). The findings of this study indicated that on the seventh day post-treatment, the PaO_2_ in the observation group increased to 92.36 ± 6.44 mmHg, compared to 88.68 ± 6.51 mmHg in the control group. The OI reached 331.84 ± 43.25 mmHg in the observation group, as opposed to 312.49 ± 41.77 mmHg in the control group. Additionally, the PaCO_2_ decreased to 41.94 ± 4.70 mmHg in the observation group, compared to 44.57 ± 4.62 mmHg in the control group. All observed differences were statistically significant (*p* < 0.05), suggesting that CPT guided by LUS can more effectively enhance the oxygenation status of patients undergoing mechanical ventilation. This effectiveness is attributed to the visual localization capability of LUS, which, through quantitative analysis of B-lines, accurately identifies areas of pulmonary consolidation. Consequently, targeted CPT measures can be implemented to facilitate airway secretion clearance, improve gas exchange, and enhance pulmonary ventilation function. The study by Huang et al. (24) demonstrated that high-frequency vibration sputum excretion enhances bronchociliary clearance function, promotes the discharge of secretions, and thereby improves oxygenation status. Longhini et al. (25) indicated that chest physiotherapy (CPT), based on the assessment of the cardiopulmonary system, employs techniques such as postural drainage, vibratory percussion, and lung expansion to improve pulmonary compliance and facilitate alveolar recruitment, thus effectively enhancing lung ventilation function. Furthermore, the study by Li et al. (26) indicated that lung ultrasound-guided postural drainage can significantly increase the oxygenation index in patients with severe pneumonia, thereby corroborating these findings.

### 4.4 Implementing CPT under the guidance of lung ultrasound can shorten the duration of mechanical ventilation and hospital stay and reduce the occurrence of complications

In terms of clinical outcomes, the observation group, in which chest physical therapy was guided by lung ultrasound, demonstrated a reduction in the duration of mechanical ventilation to 7.03 days and a decrease in the length of ICU stay to 11.62 days, both of which were statistically significant improvements compared to the control group (*p* < 0.05). Complication analysis revealed a 14% reduction in the overall incidence of complications, such as ventilator-associated pneumonia, in the observation group (all *p* < 0.05). Specifically, the incidence of VAP decreased by 6%, and the incidence of atelectasis decreased by 4%. The use of lung ultrasound-guided chest physical therapy allows for the adjustment of therapeutic plans based on ultrasound findings, significantly enhancing the patient’s ability to expectorate and facilitating sputum drainage. Previous research (27) has indicated that reducing the duration of mechanical ventilation, in conjunction with lung ultrasound monitoring of diaphragmatic thickening rate, aids in maintaining respiratory muscle function and diminishes the risk of barotrauma. With chest physical therapy guided by lung ultrasound, the physical therapy plan can be adjusted according to the lung ultrasound signs, which can significantly improve the patient’s ability to expectorate and promote sputum drainage. A study pointed out (28) that shortening the duration of mechanical ventilation and combining it with lung ultrasound monitoring of the diaphragmatic thickening rate helps maintain respiratory muscle function and reduces the risk of barotrauma. The proposed mechanism suggests that ultrasound guidance enhances the efficiency of secretion clearance and promotes lung ventilation, thereby facilitating the weaning process from mechanical ventilation and mitigating lung injury. Nicklas et al. (28) demonstrated that exercise can augment the activity of endogenous plasmin, leading to the dissolution and regression of deep vein thrombosis (DVT). Additionally, another study indicated that early functional exercise in patients undergoing invasive mechanical ventilation can decrease the incidence of ventilator-associated pneumonia and enhance the capacity for activities of daily living (29), aligning with the findings of the present study. In this investigation, the overall incidence of complications associated with invasive mechanical ventilation was significantly lower in the observation group compared to the control group (*p* < 0.05). Consequently, chest physical therapy guided by lung ultrasound not only improves the oxygenation function in patients receiving invasive mechanical ventilation but also effectively reduces the occurrence of related complications.

## 5 Conclusion

This study demonstrates that chest physical therapy, guided by LUS, can effectively enhance pulmonary ventilation and diaphragmatic function in patients undergoing mechanical ventilation, thereby improving oxygenation status. Furthermore, it significantly reduces the duration of ICU stays and decreases the risk of ventilator-associated complications. This study provides an objective and visualized approach for the assessment and intervention of respiratory management in ICU patients. However, as a single-center study with a small sample size, the research has certain limitations. Ultrasound findings are influenced by factors such as chest wall bony structures and patient body weight, which may prevent a comprehensive evaluation of the entire lung. Additionally, the study did not observe lung conditions beyond 7 days of intervention, patient outcomes after ICU discharge, or long-term quality of life after hospital discharge, which may further constrain the validity of the conclusions. Moreover, the accuracy and scientific validity of lung ultrasound scores have also been questioned in the field of neonatology (30). Therefore, future large-scale clinical studies are needed to provide further substantiation of these findings.

## Data Availability

The original contributions presented in the study are included in the article/supplementary material, further inquiries can be directed to the corresponding authors.
